# Protective effect of intensive glucose lowering therapy on all-cause mortality, adjusted for treatment switching using G-estimation method, the ACCORD trial

**DOI:** 10.1038/s41598-023-32855-3

**Published:** 2023-04-10

**Authors:** Maryam Shakiba, Maryam Nazemipour, Nasrin Mansournia, Mohammad Ali Mansournia

**Affiliations:** 1 Cardiovascular Diseases Research Center, Guilan University of Medical Sciences, Rasht, Iran; 2 Department of Biostatistics and Epidemiology, School of Health, Guilan University of Medical Sciences, Rasht, Iran; 3 Department of Epidemiology and Biostatistics, School of Public Health, Tehran University of Medical Sciences, PO Box: 14155-6446, Tehran, Iran; 4 Department of Endocrinology, AJA University of Medical Sciences, Tehran, Iran

**Keywords:** Cardiology, Endocrinology, Medical research

## Abstract

Previous analysis of the action to control cardiovascular risk in diabetes showed an increased risk of mortality among patients receiving intensive glucose lowering therapy using conventional regression method with intention to treat approach. This method is biased when time-varying confounder is affected by the previous treatment. We used 15 follow-up visits of ACCORD trial to compare the effect of time-varying intensive vs. standard treatment of glucose lowering drugs on cardiovascular and mortality outcomes in diabetic patients. The treatment effect was estimated using G-estimation and compared with accelerated failure time model using two modeling strategies. The first model adjusted for baseline confounders and the second adjusted for both baseline and time-varying confounders. While the hazard ratio of all-cause mortality for intensive compared to standard therapy in AFT model adjusted for baseline confounders was 1.17 (95% CI 1.01–1.36), the result of time-dependent AFT model  was compatible with both protective and risk effects. However, the hazard ratio estimated by G-estimation was 0.64 (95% CI 0.39–0.92). The results of this study revealed a protective effect of intensive therapy on all-cause mortality compared with standard therapy in ACCORD trial.

## Introduction

Cardiovascular disease (CVD) and premature death are among the main unfavorable outcomes among patients with type 2 diabetes. The glycated hemoglobin level, as an indicator of the mean blood glucose level, in the past 2 or 3 months has been shown to be associated with these outcomes^1,2^. In the action to control cardiovascular risk in diabetes (ACCORD) study, it was shown an increased risk of all-cause mortality in patients receiving intensive therapy of hyperglycemia compared to the standard therapy^3^. The authors used Cox proportional hazard model adjusting for baseline confounders according to the intention-to-treat (ITT) principle. However, individuals may deviate from the randomly assigned treatment at any visit by switching to other arms as a result of study protocol for threshold level of HbA1c defined per group. The dose was intensified or a new drug combination was added if HbA1c levels were ≥ 6% in the intensive group or > 8% in the standard glycemic control group, and also it was reduced if HbA1c persistently decreased to < 7% in case of hypoglycemia^4^. In such a setting in the presence of noncompliance with the assigned treatment, subsequent to switching to other treatments, the ITT approach may underestimate the treatment effect^5^. Moreover, adjusting for the noncompliance reasons at any time, influenced by prior treatment, using standard adjustment methods such as regression analysis may result in biased estimates of treatment effect due to over-adjustment and selection biases^5–14^. This condition holds in ACCORD trial, as the treatment received at visit K was determined according to the level of HbA1c in that visit which itself was affected by the previous antiglycaemic treatment^15^. Adjusting for noncompliance using causal methods such as G-estimation, inverse probability weighting (IPW), and G-formula does not introduce bias^16–28^. So, the aim of this study was to estimate the effect of intensive treatment by glucose lowering drugs on all-cause mortality using G-estimation of structural nested accelerated failure time model (SNAFTM) in ACCORD study.

## Methods

Our study was a secondary analysis of the ACCORD trial, the protocol of which has been described elsewhere^29^. Briefly, the ACCORD trial was a multicenter randomized clinical trial with a 2 × 2 factorial design in type 2 diabetic patients to investigate whether intensively targeting hyperglycemia, dyslipidemia, and elevated blood pressure can reduce the risk of CVD compared to the standard control. An informed consent was obtained from all subjects and/or their legal guardian(s). All methods were performed in accordance with the relevant guidelines and regulations. The current analysis was performed to the subset of glucose lowering therapy that was performed on all patients at baseline. A total of 10,251 patients with mean age of 62.2 (SD = 6.64) years and median HbA1c levels of 8.1% (IQR = 1.3%) were randomly assigned to the intensive or standard glucose lowering therapy. Participants in the intensive glycemic control group were treated with at least two glucose lowering medications to target a glycosylated hemoglobin (HbA1c) level < 6%, and participants in the standard glycemic control group were targeted for HbA1c levels of 7.0–7.9%. In both groups, the treatment was adjusted in case of hypoglycemia, side effects or contraindications. In the standard group, therapy was intensified in case of HbA1c ≥ 8% or reduced if persistently decreased to < 7%. The primary outcome was cardiovascular events as a composite of nonfatal myocardial infarction, nonfatal stroke, or death from cardiovascular causes. Secondary outcomes were death from any cause. Participants were followed monthly for the first 4 months and then bimonthly in the intensive and standard group until loss to follow-up, competing risks (death from other reasons), primary outcome event, or the end date of the study (July 2003), whichever came first. Physical examination and laboratory data were collected at various visits but at least at baseline and every 2 years.

### Treatment and confounders

This study was confined to 15 visits out of 90 follow-up visits which included most of the observations. The time-varying treatment was either intensive or standard therapy at each visit. As clearly has been stated in ACCORD trial, the study compared two different treatment strategy (i.e. intensive vs standard therapy) rather than two medications. The intensive glycemic control group started on > or = 2 classes of antiglysemic agents and the doses were monthly intensified if HbA1c levels were > or = 6% or if > 50% of premeal or postmeal capillary glucose readings were > 5.6 mmol/L (100 mg/dL) or > 7.8 mmol/L (140 mg/dL), respectively. In the standard glysemic control, HbA1c level was targeted at 7–7.9% and therapy was intensified whenever HbA1c was > or = 8%, or reduced if HbA1c persistently decreased to < 7% in the setting of hypoglycemia. In this study, the intensive therapy was operationally defined as receiving at least two glucose lowering treatments, and standard therapy was defined as receiving one or no treatment^4^. Age, sex, education level, race, cigarette smoking, alcohol consumption, diabetes duration (years from diagnosis), and CVD history were considered as baseline (time-fixed) confounders. Fasting plasma glucose (FPG), HbA1c, systolic and diastolic blood pressure, and lipid profile including cholesterol, HDL, LDL, and triglyceride were considered as time-varying confounders.

### Causal diagram and Statistical analysis

**Figure 1:**
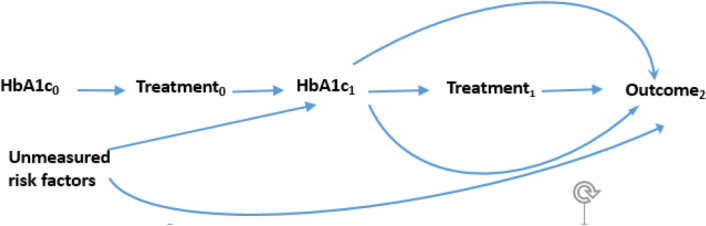
Causal diagram for the effect of time-varying treatment on cardiovascular or mortality outcome.

Figure 1 depicts the dilemma behind estimating causal effect of treatment at baseline on outcome in presence of time-dependent confounders that is affected by the previous treatment. For simplicity, only two visits and one measured confounder are shown. Subscript values imply the visit number. HBA1c is a time-varying confounder in the relationship between treatment at time 1 and cardiovascular outcome. The arrows from treatment_0_ to HBA1c_1_ suggest that HBA1c as a time-varying confounder at visit 1 is affected by previous treatment status. Adjustment for HbA1c_1_ that is also a common effect (collider) for treatment0 and unmeasured risk factor using regression models preclude unbiased causal effect estimation of treatment at baseline^6,9^. G-estimation of the SNAFTM with a two-step procedure was used to estimate the causal effect of intensive vs standard treatment by appropriately adjusting of HbA1c and other time-varying confounders affected by previous treatment values. The first step, which contained the causal variable of interest $(\varphi^{*} )$, using following formula the counterfactual failure time under no-treatment during the study denoted as $T_{{\overline{0}}}$ linked to the weighted sum of time spent with a given treatment status *A*_*k*_.

$$T_{{\overline{0}}} = \mathop \sum \limits_{k = 1}^{n} \exp (\varphi^{*} A_{k} )\Delta t_{k},$$

where A_k_ = 1 if the subject were on intensive therapy at visit K or 0 if the subject is on standard therapy. At the second step, a pooled logistic regression for receiving intensive treatment at each visit was modeled as a function of time-fixed confounders (C_0_), past values of time-varying treatment (A_k−1_) and confounders (C_k−1_), current value of confounders (C_k_), and counterfactual outcome $(\varphi^{*} )$.

$${\text{Logit}}({\text{Pr}}\, (A_{k} = 1)) = \beta_{0k} + \beta_{1} A_{0} + \beta_{2} A_{k - 1} + \beta_{3} C_{0} + \beta_{4} C_{k - 1} + \beta_{5} C_{k} + \beta_{6} T_{{(\varphi^{*} )}} .$$

This step generally stimulates randomized assignment of treatment at each visit within each stratum of previous covariate values. The two steps iteratively search for different coefficient values of causal variable $(\varphi^{*} )$ which make the treatment at each visit independent of counterfactual failure time given past treatment and confounders history. In this case it is happen when $\beta_{6} = 0$. In fact, at each visit, given the fundamental assumption of no-unmeasured confounding, we stimulate randomized assignment of treatment that is independent of counterfactual failure time. Moreover, the process of assigning treatment is conditional only on current and past values of confounder and treatment status, so eliminate the bias resulting from over adjustment of intermediate variables.

**Figure 2:**
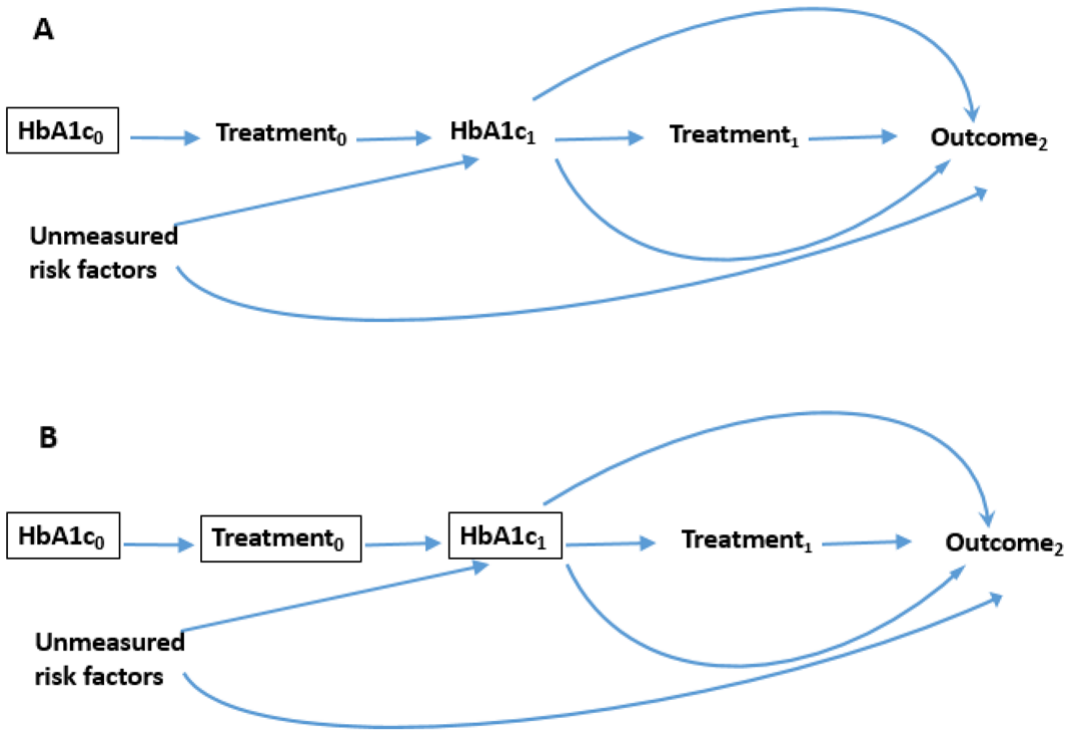
The process of adjustment in G-estimation. (**A**) Treatment at visit is generated based on current value of confounders. (**B**) Treatment at visit is generated conditional on current and past values of confounder and treatment trajectories.

Figure 2 represent the adjustment scenario in G-estimation. In Fig. 2A, the treatment at baseline is stimulated conditional on past (not shown) and current values of confounders. In Fig. 2B, the treatment at visit 1 is generated based on current and all past values of treatment and confounders’ histories.

The analysis also adjusted for loss to follow-up using inverse probability weighting of uncensored subjects. We also fitted conventional Weibull accelerated failure time (AFT) model using two modeling strategies: the first model adjusted for baseline confounders and the second model adjusted for both baseline and time-varying confounders. The results of AFT models were reported as hazard ratios with 95% confidence intervals (CIs) and the result of G-estimation was reported as hazard ratio with bootstrap-based 95% CI^30,31^. A pooled logistic regression model was used with noncompliance as a response variable, and time-fixed and time-varying confounders as well as visit as predictors. Treatment noncompliance was defined at each visit as the alteration of the treatment arm from the randomly assigned treatment at baseline based on the number of glucose lowering drugs. The conventional models were performed in Stata version 13 (StataCorp, College Station, Texas). G-estimation was conducted by SNFATM macro^24,26^ in SAS version 9.2 (SAS Institute Inc., Cary, North Carolina).

## Results

Among 9896 participants with at least one follow-up visit during a median follow-up of 4.2 years (min: 0.05, max: 4.5 years, 35,583.9 total person-years), 847 primary outcomes including 524 MI or stroke and 323 CVD mortality occurred. The incidence rate of fatal or non-fatal cardiovascular outcomes was 285 in the standard and 221 in the intensive therapy per 10,000 person years, respectively. The rate of all-cause death was 301 per 10,000 person-years in the standard therapy as compared with 134 per 10,000 person-years in the intensive therapy. The rate of CVD mortality was 13 per 10,000 person-year (22.3 per 10,000 in the standard vs 10.4 per 10,000 in the intensive therapy).

Overall, 6543 participants (64.1%) had at least one noncompliance of assigned treatment during their visits (82.4% in the standard vs 45.9% in the intensive group). Table 1 compare demographic characteristics and treatment assignment status according to compliance to the assigned treatment. The compliers and non-compliers had similar age, race, and education distribution. But there were differences in terms of sex, history of CVD, and Treatment assignment at baseline. The percentage of female, having previous CVD, and being assigned to standard group in the non-compliers was higher than compliers.

**Table 1:** Distribution of baseline demographic and treatment assignment status among complier and non-complier participants.

	Compliance	Non-compliance
Total number	6546	3662
Age (years), mean (SD)	62.6 (6.6)	62.8 (6.6)
Female sex	37.0	39.5
Race		
Black	18.5	19.5
Hispanic	7.1	7.2
Other	12.1	11.0
White	62.2	62.3
Education level		
< High school	15.6	14.3
High-school graduate	25.0	27.1
Some college	33.0	32.7
College degree or higher	26.4	25.9
CVD history at baseline	33.7	35.9
Treatment assignment at baseline		
Standard	24.5	64.0
Intensive	75.5	35.9

Values are percent unless otherwise indicated.

The noncompliance of treatment protocol per visit was also different between the two groups. The rate of noncompliance was 66.4% (41,239 person-visits) in standard therapy group as compared with 16.7% (10,341 person-visits) in the intensive-therapy group (P-value < 0.001), while there was no substantial difference in the loss to follow-up between the two groups (7.4% and 7.5% in the standard and intensive therapy, respectively).

Table 2 shows important predictors of noncompliance in the two groups. Baseline HbA1c (OR = 1.18, 95% CI 1.09–1.28) and lagged values of HbA1c (OR = 1.46, 1.32–1.60) increased the odds of noncompliance in the standard therapy, while female sex and CVD history was associated with increased odds of noncompliance in the intensive therapy.

**Table 2:** Adjusted odds ratios (95% confidence intervals) of treatment noncompliance by treatment arm.

	Standard therapy	Intensive therapy
Age (years)	0.98 (0.97–0.99)	1.03 (1.10–1.61)
Female sex	0.80 (0.74–0.87)	1.29 (0.92–1.82)
Race		
Black	Reference	
Hispanic	1.23 (1.02–1.48)	0.35 (0.15–0.77)
Other	0.98 (0.85–1.14)	0.53 (0.28–1.02)
White	1.22 (1.09–1.36)	0.97 (0.43–1.29)
Education level		
< High school	Reference	
High-school graduate	1.01 (0.88–1.16)	1.25 (0.74–2.12)
Some college	0.91 (0.80–1.04)	0.68 (0.39–1.16)
College degree or higher	1.18 (1.02–1.35)	0.75 (0.43–1.29)
CVD history at baseline	0.82 (0.75–0.89)	2.03 (1.46–2.83)
Years lived with diabetes	0.99 (0.98–0.99)	1.04 (1.02–1.06)
Fasting plasma glucose at baseline	1.00 (1.00–1.003)	0.99 (0.99–1.00)
Fasting plasma glucose at current visit	1.00 (0.99–1.00)	1.00 (1.00–1.004)
HbA1c at baseline	1.10 (1.05–1.16)	0.94 (0.77–1.15)
HbA1c at current visit	1.04 (0.97–1.10)	1.12 (0.86–1.45)
HbA1c at previous visit	1.37 (1.29–1.44)	0.89 (0.69–1.15)

Table 3 shows the results of conventional regression analysis using two modeling strategies and G-estimation and compared them with the result of the primary ACCORD trial^32^. The first conventional regression model adjusting for only baseline confounders showed a 17% (95% CI 1–36%) increased risk of mortality in the intensive therapy compared to the standard therapy. The results of the second model adjusting for both baseline and time-varying confounders were compatible with both protective and risk effects for CVD as well as all-cause and CVD mortality^33,34^. G-estimation showed that continuously treatment by intensive therapy decreased the hazard of mortality by 36% (HR = 0.64, 95% CI 0.39–0.92).

**Table 3:** Hazard ratios of CVD outcome, all-cause, and CVD mortality for intensive compared to standard therapy in the ACCORD trial.

	Cardiovascular outcome	All-cause mortality	CVD mortality
The ACCORD trial result (baseline adjusted Cox model)	0.90 (0.78–1.04)	1.22 (1.01–1.46)	1.35 (1.04–1.76)
Baseline adjusted Weibull model	0.89 (0.79–1.02)	1.17 (1.01–1.36)	1.26 (1.01–1.58)
Time-varying adjusted Weibull model	0.49 (0.22–1.06)	0.34 (0.10–1.10)	0.33 (0.06–1.87)
G-estimation	0.89 (0.43–2.26)	0.64 (0.39–0.92)	0.71 (0.29–1.24)

## Discussion

In this study, we estimated the effect of time-varying intensive glucose lowering therapy vs. standard therapy among type 2 diabetic patients on cardiovascular and all-cause mortality outcomes using the G-estimation and compared it with conventional regression model. We used this method because conventional methods may result in biased effect estimates when there are time-varying confounders affected by prior treatment. In ACCORD trial, non-compliance was high especially in the standard therapy group, and treatment at each visit (after baseline) was influenced by previous values of HbA1c. Specifically, the patients with higher baseline and lagged values of HbA1c in standard therapy were more likely to give up their assigned treatment. In such situations, conventional methods may result in biased effect estimates because of over-adjustment and selection biases^35–40^ when the reasons for noncompliance at any time are affected by unmeasured risk factors of the outcome and prior treatment received^6,19^. The G-estimation results revealed a significant effect of intensive therapy on reducing the risk of all-cause mortality that was in contrast with the result of ITT approach using conventional regression analysis adjusted for baseline variables in current study and previous result of ACCORD trial^32^. Both baseline adjusted model either as AFT approach in the current study or Cox proportional hazard model in the primary study of ACCORD^32^ gave almost similar results for both outcomes. These results cannot estimate the causal effect of treatment because the treatment received at each visit after baseline is a variable varying over time based on the patients’ profile including HbA1c. In randomized trials, the effect estimate of treatment using ITT approach would be unbiased if all subjects in each arm take their treatment at all times and are under complete follow-up throughout the study. In the ACCORD trial, the percent of loss to follow-up was trivial but the noncompliance percent was high with a substantial difference between the two treatment groups. On the other hand, the main drawback of conventional ITT regression approach is that it estimates the effect of randomly assigned treatment, but not the received treatment at each visit, which is not of interest^19^.

The estimates obtained from time-dependent model are also subject to selection and over-adjustment biases because of inappropriate adjustment of time-varying confounders. G-estimation overcomes this deficiency by estimating the effect of received treatment under the assumption of sequential randomization (conditional exchangeability) at each visit, given the measured confounders. The effect of interest is the effect that would have been observed if all patients in the trial had compliance to the study protocol^14^. The selection bias induced by censoring was also adjusted by IPW of uncensored subjects.

The G-estimate in the current study indicated protective effect of treatment on mortality, but failed to show the same effect on CVD. This might be explained in part by the association of HbA1c with all-cause mortality that has been reported by several recent meta-analyses and large-scale observational studies^41–44^. In a meta-analysis of observational studies, both higher and lower levels of HbA1c had significant association with all-cause and cardiovascular mortality in diabetic patients but no association was found with cardiovascular events^42^. Similarly, another meta-analysis of observational studies showed a significant J shape relationship between HbA1c and all-cause mortality^41^. In a nationwide, community-based cohort study, the highest risk of all-cause mortality was found for HbA1c level < 5.6% or > 7.4% compared to 6.5%^43^. In this study, HbA1c was a strong time-varying confounder and significant predictor of noncompliance in the standard treatment group indicating that the patients in the standard group were more likely to withdraw their assigned treatment and switch to intensive treatment. On the other hand, switching to standard treatment was less likely to occur in the intensive group. These conditions remind the importance of appropriate adjustment for time-varying confounders which cannot be estimated using conventional regression analysis.

G-estimation has been applied in a number of observational studies^45–50^, but a few randomized trials used this methodology to estimate treatment effect in the presence of non-compliance^51,52^. RCTs are frequently analyzed according to the assigned treatment at baseline as either ITT or per protocol (PP) analysis. The former approach aims to preserve the original randomization and the latter excludes those patients that are not fully compliant^53^. But, post-randomization events affected by treatment invalidates PP analysis^54–56^. In RCTs, in the absence of loss to follow-up and noncompliance, the ITT approach unbiasedly estimates the causal effect of interest^19,52^. Given the inevitable occurrence of noncompliance in every RCT, the ITT approach with conventional regression method that is often the only primary analysis is recommended to be replaced by modern methods accounting for noncompliance and time-varying confounding such as G-estimation^52^.

## Conclusion

Adjustment for treatment switching using the method of G-estimation revealed a protective effect of intensive therapy an all-cause mortality compared with standard therapy in ACCORD trial that was in contrast with the result of conventional regression analyses and ITT approaches published before.

## Data Availability

The data that support the findings of this study are available from [The National Heart, Lung and Blood Institute (NHLBI) Biologic Specimen and Data Repository] but restrictions apply to the availability of these data, which were used under license for the current study, and so are not publicly available. Data are however available from the first author (Maryam Shakiba) upon reasonable request and with permission of [The National Heart, Lung and Blood Institute (NHLBI) Biologic Specimen and Data Repository].
